# Leishmaniasis and Climate Change—Case Study: Argentina

**DOI:** 10.1155/2012/601242

**Published:** 2012-05-20

**Authors:** Oscar Daniel Salomón, María Gabriela Quintana, Andrea Verónica Mastrángelo, María Soledad Fernández

**Affiliations:** ^1^Instituto Nacional de Medicina Tropical, Neuquén y Jujuy, Puerto Iguazú, Consejo Nacional de Investigaciones Científicas y Técnicas CONICET CP3370, Argentina; ^2^Instituto Superior de Entomología “Dr. Abraham Willink”, CONICET Miguel Lillo 205, San Miguel de Tucumán CP4000, Argentina; ^3^Universidad Nacional de Misiones, CONICET Posadas CP3300, Argentina; ^4^Centro Nacional de Diagnóstico e Investigación de Endemo-Epidemias, CONICET Paseo Colón 568, 1er piso, Ciudad de Buenos Aires, UBA CP1063, Argentina

## Abstract

Vector-borne diseases closely associated with the environment, such as leishmaniases, have been a usual argument about the deleterious impact of climate change on public health. From the biological point of view interaction of different variables has different and even conflicting effects on the survival of vectors and the probability transmission of pathogens. The results on ecoepidemiology of leishmaniasis in Argentina related to climate variables at different scales of space and time are presented. These studies showed that the changes in transmission due to change or increase in frequency and intensity of climatic instability were expressed through changes in the probability of vector-human reservoir effective contacts. These changes of contact in turn are modulated by both direct effects on the biology and ecology of the organisms involved, as by perceptions and changes in the behavior of the human communities at risk. Therefore, from the perspective of public health and state policy, and taking into account the current nonlinear increased velocity of climate change, we concluded that discussing the uncertainties of large-scale models will have lower impact than to develop-validate mitigation strategies to be operative at local level, and compatibles with sustainable development, conservation biodiversity, and respect for cultural diversity.

## 1. Introduction

Insect-borne diseases, especially those closely associated with natural environments, such as malaria or leishmaniasis, have been frequently cited as an argument on health risks related to the change and instability of the climate.

However, many of these statements far from predictions based on experimental data have added a new dose of uncertainty in already uncertain models and conjectural discussions [1–4]. Considering only the biological aspects at individual level, the effects of the climate changes and instability on vector-borne diseases transmission may be the consequence of multiple variables such as daily and mean, maximum and minimum temperature, amount of days with temperature above a certain threshold, relative humidity at different times of the day and across the seasons, accumulated precipitation in different periods prior to the date of interest, soil moisture, and changes associated with human use of land. The effect of these variables is more difficult to follow accurately when they are looked at as interactions between each other and their impact on the environment, which modulates the vector metabolism, survival, and daily activity of larvae and adults (success of trapping expressed sometimes as abundance), the infectious period, the extrinsic incubation period, and the number of infectious events [5–9]. Thus, according to the relative weight given to each of the different climate-related variables and the way that could affect each stage of the vector, it can be projected an increase or decrease of parasite transmission, and sometimes opposite trends simultaneously, which has led to academic controversies [10–13]. The problem became even further complex at the level of populations or communities, including parasites, vectors, reservoirs, and hosts (among them humans) distributed dynamically in space and time in a matrix of changing environment [14–16]. Despite noted difficulties, discussion section deals with many attempts which were made for leishmaniases in different time and space scales.

These words of caution should not be understood as skepticism. However, climate change is an ongoing fact [17, 18], and today we look for strategies that in the short-term could mitigate the climate change effects at operative scales may have more impact from the public health point of view, than to discuss theoretically the global models of climate change.

Therefore, taking into account the network of causes and results, in order to obtain a probable projection on actual and specific scenarios, the impact assessment on collective health of climate change, biodiversity conservation, and/or sustainable development [19] requires the epistemological framework of the ecoepidemiology [20, 21]. To do so it is necessary (1) to address the consistency between assumptions, indicators, analysis, and conclusions and validate the consistency with the units of reference, its space-time scale of resolution, the quality of data sources, and analytical method and (2) to integrate social variables with the biological ones involved in change scenarios [2, 8, 22–25].

To illustrate the two statements mentioned the research on leishmaniasis in Argentina is presented as a case study.

## 2. Leishmaniases in Argentina

Reported since the beginning of the XX century, American cutaneous leishmaniasis (ACL) has significantly increased its incidence in Argentina during the 1980s decade, while the first human recorded autochthonous case of visceral leishmaniasis (VL) was diagnosed during 2006 [26, 27].

**Figure 1:**
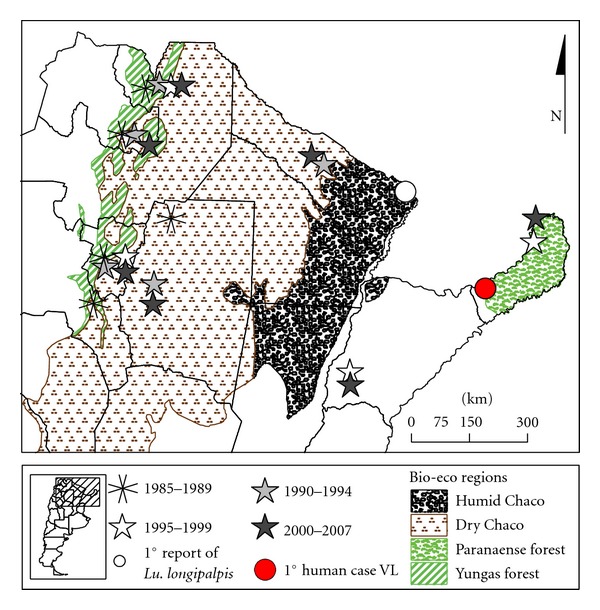
Leishmaniasis in Argentina by bio-eco regions. Distribution of outbreaks by site and period.

Endemic in ten Northern provinces and four bioecological tropical/subtropical regions (Foothills of Yungas Forest, Dry Chaco, Wet Chaco, and Paranaense Forest) (Figure 1), the cases of ACL were usually isolated in time and space or related to a “common source” like a punctual deforestation up to the decade of 1980. The reports by year usually fluctuate between 40 and 90 for the whole country. However since 1985 the cases clustered in outbreaks up to 900 cases, beginning in the northwest (Yungas) and following in the next years by new outbreaks to the east, reaching the Eastern border of the endemic area by 1998 [28] (Figure 1). The last epidemic year was 2002 with 748 human cases throughout the ten endemic provinces (rate of incidence/year 0.2/10 000 inhabitants), and since then it began up to now an interepidemic period with smaller foci sparse in the whole endemic area (150–370 cases). As the usual agent of ACL present in the country is also related to mucocutaneous leishmaniasis (*L. braziliensis*) the decrease of incident cutaneous cases, and the past cutaneous epidemics, resulted in an increase in the ratio of mucosal to cutaneous cases (2010 : 28 : 138) (SNVS National System of Surveillance, Ministry of Heath).

Three species of *Leishmania *have been isolated from humans with ACL in the country, *L. (Viannia) braziliensis, L. (Leishmania) amazonensis*, and *L. (Viannia) guyanensis*, and from VL cases *L. (Leishmania) chagasi*. Of these, *L. braziliensis* has been the main agent associated with ACL outbreaks and with mucosal involvement. The reservoir of *L. braziliensis* remains undefined although many mammals (e.g., Canidae, Equidae, and rodents) are naturally infected; no one animal completely fulfills the requirements to be defined as the single reservoir [29]. Since the first reported outbreak of ACL in Argentina in 1985–87 (Figure 1), 28 different species of Phlebotominae have been reported. Among these, *Nyssomyia neivai, Ny. whitmani, Mygonemyia migonei*, and *Evandromyia cortelezzii-sallesi *have been incriminated as vectors of *L. braziliensis *[30, 31].

**Figure 2:**
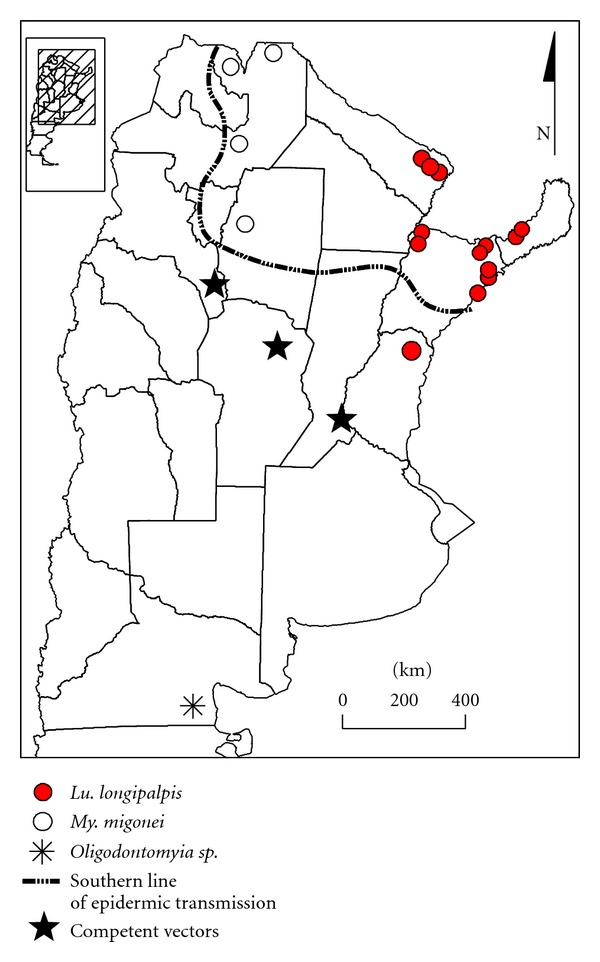
*Lutzomyia longipalpis* spread (black circles) and human cases associated with *My. migonei* as its putative vector (white circles). Southernmost record of Phlebotominae and records of competent vectors.

The first known report of a human case of VL, with infected dogs and the vector *Lu*. *longipalpis *in its urban courtyard, was reported in Posadas, Misiones, in northeastern Argentina in 2006 (Figure 1), probably infected during the previous year [27]. However, the vector was found in urban environments since 2004 in the Paraguay-Argentina border, in the province of Formosa [30] (Figure 1). Most of the human cases are still from the province of Misiones, with *L. infantum* (*syn. chagasi*) isolated from humans, dogs, and vectors [32], but the vector of VL spread to the south and the west of the country, in three provinces (Corrientes, Entre Ríos, Chaco), and human cases were recorded in Corrientes province, at the south of Misiones province [31, 33, 34] (Figure 2). In the western area of the country sparse human cases (Santiago del Estero and Salta provinces) were associated with *My. migonei* as its putative vector [35, 36] (Figure 2). The accumulated VL cases reported from 2006 to July 2011 are 81, while dogs infected by *L. infantum* were reported in almost all the country due to the social and commercial networks related to dog relocation (SNVS National System of Surveillance, Ministry of Heath).

## 3. Materials and Methods

Captures of vectors of leishmaniases were performed with minilight traps CDC-like operating overnight and Shannon traps. The design of the captures varies covering different extensions of space and periods of time according to the objectives of each particular study. The captures were made by the researchers of the Argentinean Network of Research in Leishmaniasis (REDILA) in 12 provinces, belonging to the three bioecological regions included in the endemic area of leishmaniases: Yungas Forest (Northwest) Catamarca, Jujuy, Tucuman, Salta; Chaco Savanna (wet and dry): Chaco, Córdoba, Formosa, Santiago del Estero; Paranaense Forest: Corrientes, Entre Ríos, Misiones, Santa Fe (Figure 1). The insects were cleared and the species assigned using the keys of Young and Duncan [37] and Galati [38]. The parasites detected in natural infections were genotypified by PCR-RFLP specific for species, with subsequent sequencing or dotblot. The variables of climate and landscape were analyzed by multiple correlation and time series ARIMA models and the spatial integration and forecasting by maximum entropy's modeling system (MaxEnt). The anthropological studies involved key informants, in-depth interviews, and participant observation. The description of the specific methodology, the criteria for the selection of the sites of capture, and the analytic strategy of each study could be found in the references cited in the following section.

## 4. Results and Discussion

### 4.1. Argentina Case Study

#### 4.1.1. Microscale

The microfocus scale refers to the events that happen within the area surrounding the sampling point with a radius that varies according to the subject of focus of the study: autonomous flight of the vector (adult), territory of the reservoir, space where the householders develop their activities, and so forth. Variables as land cover, land use, and extreme weather events such as floods to model species distribution become usually more important on smaller spatial scales [25, 39, 40].

In this scale the variables associated with the climate, as the low temperatures, mainly the winter in the latitudes with distinct seasons it was suggested as a period with no adult activity from vectors of leishmaniases. However, competent vectors have been captured during temperate winter nights in microhabitats protected from sudden climate changes, as primary forest remnant patches [41], or habitats that moderate these changes as patches of secondary vegetation [42]. Thus, suggesting even a small increase in temperature could lead to continuous generations of vectors and increased risk of parasite transmission at “hot-spots.” But this increase in temperature should have a minimal range, as isolated warm nights probably do not provide the females time enough to have a second bloodmeal, the former to get the infection and the second one to transmit it. On the other hand, if the accumulated temperature/days (ADD) [5] allows the parasite cycle to be completed, so the period of active transmission could be longer than that observed during a regular year.

In this sense, environmental management, or just any anthropic or climate-driven changes in the landscape, can generate or destroy these temporary shelters of vectors. This fact is even more important for artificial refuges, such as those built for breeding domestic animals, with temperature and humidity favorable for the sand flies, and continuous source of food (for larvae: organic matter in soil; for adults: blood). Shelters, in turn, are associated with greater abundances of vectors in the edge of deforestation—cluster analysis [43], as a local population from a source population located within a dense vegetation patch cross-correlation/metapopulation structure [44], or consequence by the deforestation itself in the interface with cultures generating a “border effect” nonmetric multidimensional scaling and Kendall correlation coefficients [45].

In relation to the abundance activity per hour, the vector of VL *Lu. longipalpis* in December (summer) in the Northeast of the country (NEA) showed a curve associated positively with the average temperature and relative humidity (RH), in the case of females. The “window” (best temperature and humidity condition) of greater abundance for females was between 26° and 28°C and 63 and 68 RH%; the 90% of the females were captured between 20:30 pm and 1:30 am [46]. In the case of *Ny. neivai* in the Northwest (NOA) although with a bimodal pattern, in January (summer) the peak of greater activity was at 00:00 am with the temperature as the variable that best explained abundance by hour and in April (autumn) at 03:00 am with the humidity as the critical variable [47]. Thus, the effect of temperature and humidity on the abundance activity of sandflies varies according to the species, with different critical variables according to the season and region.

Furthermore, taking into account the micro focus biological frame, an increase in temperature or humidity, preserving the light-darkness period, would not only change the transmission risk by the duration of night activity of the females but also could be accompanied by changes of the habits of humans. This human behavior changes would increase the vulnerability discriminated by socioeconomic variables. For instance, in very hot and humid nights (more prolonged activity of vectors) some families spend more time in the courtyard during and after the supper, while other families spend this period indoors with air conditioning or roof fans, increasing so the inequity in the distribution of health events.

#### 4.1.2. Mesoscale

The mesofocus scale includes the population-based studies, such those on epidemic outbreaks or populations of vectors and/or reservoirs, and is the more frequent scale of study of classical epidemiology.

 In this scale many Phlebotominae species in several foci presented peaks associated with the periods of rain [45, 48]. However it should be distinguished between the emergence peaks of young adult vectors and the period with higher probability of transmission of leishmaniasis, with peaks with less amount of individuals but with more older ones (females with at least one intake and therefore more likely to have ingested parasites) [44]. Therefore, the peaks of cutaneous leishmaniasis according to the probable date of start of the lesion (ACL) are associated with the peaks of gravid females, and not with the highest peaks of adults [28]. It means that given the incubation period of leishmaniasis in humans, the increase in cases is usually recorded in epidemics or during endemic years, for ACL and VL, towards the end of the season of activity of vectors, in Argentina during autumn, especially in the month of April [49–51]. Consistently with these results, the best combination of climatic predictors of abundance of Phlebotominae usually includes rain [52], and the vectors peak is positively associated at different times after rainfall, with lag periods of rainfall-vector abundance up to one year for *Ny. neivai* [28, 44, 53].

As climate phenomenon the rainfall is associated with water balance and runoff coefficient, shadow-roofed protection available, and relative humidity temperature. These effects modulated by the precipitation and temperature would have in turn impact on the larval substrate, where a “window” of moisture of the soil is necessary for the survival, different effects from that of the weather variables as metabolic velocity modulators on larvae but also on adults. This differential effect by stage was suggested from what was observed for* Ny. whitmani* and *My. migonei* in an endemic of ACL area, recently deforested. There, the vector abundance was associated positively with the temperature at the time of capture (day 0) and 30–45 days before the sampling but with the rain only showed a positive significant association 30 days before the capture. The association with rainfall disappears when the effect of temperature is removed, which would indicate that in an area without dry season the temperature becomes the critical variable [54]. These delayed effects of the abiotic factors on the sand fly population were also reported for *Lu. longipalpis* in a VL focus of Brazil [55]. These variables, in turn, could play different roles in source/sink populations of a metapopulation structure, as it was observed for ACL vectors [28, 42, 44], and VL vectors [56], that in Posadas focus, had a spatial autocorrelation of 600 m [57, 58]. The weather-associated variables together with the anthropic environmental and animal management even could generate a dynamic metapopulation with “hot spots” alternating in time its role as source or sink populations.

#### 4.1.3. Macroscale

The macro focus scale refers to regional and subcontinental phenomena; it is the usual scale of projective and predictive climate-based models. When it is used to explain epidemiologic facts at meso- or microscales, it can distort the forecasts, by compensation of field-data with opposite trends or by magnifying the data taking only in account the more visible events (endemic pattern analyzed using epidemic records). Therefore, alterations in rainfall would have a direct effect on the regulation of the populations of Phlebotominae, and although climate change affect large regions (macroscale) in each one (focus scale) could result in a different scenario of transmission, clustered in time and space (microfocal scale). So, for instance, high instability due to El Niño South Oscilation (macroscale) increases or decreases the incidence of leishmaniases with extraordinary rainfalls and drought at different foci (meso-scale). The actual distribution of “hot spots” with increased probability of transmission during excessive rains could be clustered in high topographic locations that remain relatively dry, while during droughts the “hot spots” could be located in shadowed depressions of the landscape that retains humidity [59].

In the frame of this scale, the current and potential distribution of Phlebotominae in Argentina was analyzed. The southernmost specimens found were of the genus *Oligodontomyia*, without known vector capacity, in the plateau of Somuncurá, located between 40°20′ and 41°30′ S and 65°55′ and 70°10′ W [60] (Figure 2). Further, the presence of *Evandromyia* individuals was reported during the early twentieth century in the Province of Buenos Aires, but never afterward when these areas underwent an intense transformation and urbanization. However, these data demonstrate the ability of these insects to be present and colonize places with usually considered adverse weather conditions. So far the southernmost current captures obtained from the competent vectors *Ny. neivai* and *My. migonei* were at coordinates 31°12′46′′ S–60°09′22′′ W and 31°35′15′′–31°35′15′′ S–60°17′48′′ W [61] (Figure 2), along the gallery forest of the Paraná river under a process of tropicalization [62].

The xerophytic bio region of the Chaco dry forest has its own vectors, associated with sporadic zoonotic transmission of ACL, *My. Migonei,* and *Ev. cortelezzii-sallesi* as prevalent species, the last found with natural infection of *L. braziliensis* [63]. However, an increase in precipitation and humidity, as it was observed in the province of Santiago del Estero both by global trends and water reservoir-irrigation development projects [64, 65], could transform the pattern of abundance/diversity towards the epidemic pattern found in the humid Chaco, the Northwest and Northeast regions with *Ny. neivai*, as the prevalent peridomestic species, followed by *My. migonei* as a vector “hinge” connecting zoonotic and anthropo zoonotic parasite transmission cycles [46, 52, 66].

Finally, when performing potential distribution models (method of MaxEnt modeling) in northwest of Argentina for *Ny. neivai* and *My. Migonei,* where the rainfall of the driest month was variable that best generalized both models [67], an increase of precipitation would increase the area of dispersion of the species.

*Lutzomyia longipalpis's* dispersion in Argentina, since the urban reports in Campo Grande, Mato Grosso do Sul, in Brazil and Asuncion, in Paraguay, was followed also in the three analytical scales. *Lutzomyia longipalpis* in Argentina was recorded by first time in 1951 in Candelaria, Misiones (one female), and again in the year 2000 in Corpus, Misiones (4 out of 9253 Phlebotominae), both rural areas with very low human density at the time or place of capture. Further, since 1990, more than 80000 Phlebotominae had been captured and registered in Misiones and the other 8 provinces endemic for ACL in Argentina, but only *Lu. longipalpis* was registered in the locality mentioned above [68]. The first record of this vector associated with urban habitats and epidemic scenarios in the country was to the end of the year 2004 in Clorinda, province of Formosa (Figure 1), just in front of the VL focus of Asunción-Lambaré, in Paraguay, across the river [30]. The first urban autochthonous focus in Argentina as it was already described in the introduction was reported in province of Misiones in June 2006 (Figure 1), with simultaneous presence of *Lu. longipalpis* and an infected dog in the courtyard of a human VL case, that probably would have been infected in the second half of 2005 [27]. *Lutzomyia longipalpis*, humans and dogs with VL were observed during the summer 2008–2009 in the province of Corrientes south to Misiones, on the shore of the Uruguay River and Parana River, including areas with intensive captures over the previous 5 years, due to transmission of ACL, but with no *Lu. longipalpis* found then [31]. In the summer 2009-2010, *Lu. longipalpis* was further dispersed to the south and it was found in Chajarí, Entre Rios (30°40–46′ S–58°00′–57°57′ W), Bella Unión and Salto, Uruguay (31°23′49.9′′ S, 57°57′50.4′′ W), and also spread westward across the Parana River to Resistencia, Chaco (27°25–28′ S–58°58–59′ W) in sites where regular captures catches had been made without previous presence of this species [33, 34, 69] (Figure 2).

The specimens of *Lu. longipalpis* found in Posadas had natural infection with *L. infantum* with a rate of at least 0.47% [32]; in order to understand its possible route of dispersion from the northern foci, the sex pheromone of the male was characterized as (S)-9-methylgermacrene-B, the same described from *Lu. longipalpis* of Paraguay and several populations of Brazil. However, the analysis of the *period *gene sequences found that the populations of Argentina are significantly different from those previously studied in Northeastern and Southeastern Brazil, although it would require further studies of intermediate locations before characterizing these “southernmost” populations of *Lu. longipalpis *as in the way to differentiation [70]. When we applied the modeling of potential distribution to *Lu. longipalpis*, with an analytic strategy similar to that used for *Ny. neivai* and *My. migonei*, we observed that the vector of VL can colonize the gallery forest of the Uruguay River, and the precipitation was the variable that best generalized model.

The potential distribution derived from the modeling shows that changes in the rainfall patterns (mean, variability, frequency, distribution in time and space seasonality, availability of sources) [71, 72] will modulate the geographical dispersion or extinction of the main vectors of ACL and VL in the large scale.

Besides, in the Chaco bioregion, sporadic cases of human VL have been associated with *My. migonei* as the putative vector of *L. infantum*, without *Lu. longipalpis*, but in a context of intensified parasite circulation (migration of infected dogs from endemic urban foci with *Lu. longipalpis* and with a growing awareness of the health system due to the outbreaks in the provinces of Misiones and Corrientes) [35]. Thus, again, an increase in humidity of the Dry Chaco region can eliminate the climate barrier to dispersal of the urban epidemic form of VL produced by *Lu. longipalpis*. This phenomenon was already proposed for ACL in the twentieth century, for two bioregions, the foothills of Yungas and central Chaco, where the increase in rainfall turned profitable the deforestation followed by human settlement for agriculture instead of just logging and abandonment of the deforested lands [73–75]. This change in human behavior driven by climate change increases consequently the likelihood of effective human-vector contact and also pushes the adaptation of the epidemic vector of ACL, *Ny. Neivai,* to the anthropic modified landscapes, which thereafter became prevalent and abundant in peridomestic rural and ruralized periurban habitats (border effect and domestic animal management).

## 5. Climate Change and Leishmaniases in the World

 The search for articles on climate change and leishmaniasis (MEDLINE 25/07/2011) recorded 25 papers of which 14 are reviews. Defined geographically 13 belongs to Europe, 3 to Latin America, 2 to Asia, and 1 to North America. The authors usually point to the need for intensified monitoring of leishmaniasis due to the risk of geographic range expansion and the intra-annual and interannual variability of incidence due to changes in distribution, abundance, and diversity of competent vectors. These projections are based on expert knowledge of prevalence distribution or biology and current distribution of the vector, overlapped to a bioregion or climatic conditions, usually temperature [76–84], only excluding the risk of latitudes as Denmark [85]. With less conjectural methods, some authors develop predictive models of potential distribution for competent vectors making approaches in the macroscale through different scenarios of expected changes in temperature [86]. With this approach for *Phlebotomus papatasi* in Southwest Asia an increase of 1°C predicts an increase of endemic sites of 14% (14 out of 115 stations considered, 71 currently endemic) if the increase reaches 3°C 15% additional sites are added, and in 7% of the stations the seasonal transmission period would be extended to the whole year [87]. In South America this analytical strategy was used to project the distributions of three species of sanitary importance, *Ny. whitmani*, *Ny. neivai* and *My. migonei*, where an increase in temperature would have more impact in the dispersion in the macro-scale, that if will including topographic data [4]. This latest variable was included for United States and Canada predicting a doubling of humans exposed to vector-borne transmission of leishmaniasis even with an occupation of the vector and reservoir of 10% of favorable habitats [88] towards the 2080. MaxEnT models with bioclimatic change scenarios at the regional level were used for central Europe, and the favorable regions were identified including the approach of least-cost path, obtained from expert information about vectors and topography. Thus, while ecological niche models develop algorithms correlating records of presences absences of a species with quantitative ecological variables, the integration of these results with alternative climate change models and species-specific dispersal ability (least-cost analysis) allows to project new potential distributions [86]. These projections show an increase in appropriate habitats for vectors since the second half of the 21st century, although the Phlebotominae would not be occupied all due to limitations in their ability to spread and the Alps as a barrier to expansion towards the North [89]. It is interesting to note that this potential for dispersal and reintroduction (depending on the scale of time used) in central Europe varies to Austria according to the species and station, so with an increase of 1°C for *Ph. mascitti* appropriate habitats would occur in January, while for *Ph. neglectus* in July [90]. Models using degree/day have been also applied to estimate the number of generations, peak activity, and annual variability in Iran [91].

The species to deal with changes in its preferred bioclimatic niches and to spread to other suitable-available habitats driven by climate change are limited by its own intrinsic biological resilience or potentiality and its population-metapopulation dynamics, but interacting with the dynamics of other coexistent species and the dynamics of fragmented landscapes. Therefore, as it has been pointed out on numerous occasions, it is very difficult to extrapolate these models outside of the area used for its construction, integrate statistical spatial models based on climate with biological models, and develop based on these models early warning systems with appropriate field-based surveillance response [38, 39, 92–95].

However, despite so many statements in lectures and documents, unfortunately the evidences of the actual impact of climate change on the transmission of leishmaniasis with field-based data are still few or weak and ever closely related with changes in human behavior. In the United Kingdom it was noted that 105 out of 183 dogs came from Spain with *L. infantum* infection. The authors highlight the risk of moving infected reservoirs to sites that can become favorable to the development of vectors and provide a possible association between the increase and canine incidence in the period 1994–2007, with other areas not experiencing the same process (55). The migration of infected pets treated with medications approved locally is also indicated as a variable of risk of dispersion within Europe due to “a flourishing market for dogs of miserable appearance suffering from leishmaniases (that) has been developed by profit-oriented opportunists” [96] and to spread of parasite strains that are resistant to drugs used for humans in other continents [97]. In this sense both the world trade of goods, humans and animals and the climate change and seasonal variability drive the pattern of dispersion, range of dissemination, amplification, and persistence in new environments of pathogens such as *Leishmania* [89, 98, 99].

On the other hand, the difficulty to understand these phenomena with events measured in different scales, as it was mentioned in the results, was also emphasized when comparing macroscale indicators, as the intensity of “El Niño” or “La Niña” with changes in the global incidence of leishmaniasis discriminated by departments/provinces/states [100], where climatic instability creates different scenarios of transmission, even with opposite sign at the same time in the mesoscale and microscale [1]. In this regard it should be taken into account the change and climatic instability along with other variables such as land use, management of water, human population growth and urbanization, chemical pollution and the movements of people, animals and property as parts of a global change, each one different in velocity and local impact, defying in a different way the adaptive capacity of each community, its regional economy, and the quality and coverage of their health systems [84, 98, 101]. Mixed scales were often seen both conceptually and in actual field designs discussing altitude range as subrogate of climate and vegetation ranges, and mean temperature or rainfall together with border effect, wind obstacles as walls, species of trees, domestic blood sources, the lacking of paved roads.

Therefore, although socioeconomic variables are more often associated with other diseases as malaria or cholera, while leishmaniasis for many studies looks like to be only affected by abiotic factors [102], it should be taken into account that the cultural factors are not an additional approach to the biological problems, but part of it [103]. This theoretical frame involves also approaches in different scales from the macroscale as the perception of climate change by researchers, media, and community [104, 105]; migrations due to drought, war, or global economic market oscillations as it was the development of intensive cocoa and rubber cultures associated with outbreaks of leishmaniasis in Brazil [82, 106, 107] the impact of climate change on local knowledge and traditional technologies [108] the dispersion of reservoirs through social and commercial networks such as dogs in the case of VL [109] and the microscale as the risk and environment perception, which is itself a risk variable, compete with the necessity when the space of daily activities (handling pets, housing, collecting water or firewood) are constructed and defined by householders [110, 111], but the consistency of scales should again be essential both to support conclusions and to generate appropriate and feasible recommendations with actual impact in the health of individuals and communities [112].

Finally the modification of the environment is also closely associated with both cultural issues as climate change, generating a cascade of events that can start with deforestation, followed by changes in land use and human settlements, habitat fragmentation, border effect, increased abundance or contact with synanthropic reservoirs, clustered food sources for vectors (domestic animals), pressure for adaptation of vectors to modified environments and anthropophily, irrigation and water storage systems and optimization of vector and reservoirs breeding conditions [113], human susceptible migration and unplanned urbanization (favorable habitats), impoverishment and immune status of the human population and deterioration of the public health systems [82, 114].

## 6. Conclusion

The impact of climate change on vector-borne diseases, as leishmaniasis, although intuitively logical and currently in an ongoing stage, requires more strong and earlier biological-based evidences, such as those obtained by long-term monitoring in transects to determine the altitudinal and latitudinal range of each species of vector [115]. However, it is also essential to be able to refute the skeptic discourse [116] to bring a strong scientific support to the climate change vector-borne disease debate, to discriminate the hypotheses data conclusions in adequate and consistent scales of time and space, and to take into account the intermingled net of physical-abiotic, biological, and social-driven variables that are playing in these changing scenarios.

In conclusion, in order to transform the knowledge into actual mitigation actions at local level it should be developed the following.

**(1) Risk Maps**

To define in each scale the spatial and temporal vulnerability of dispersión colonization of vectors/outbreak of disease, by assessing the risk variables (biomedical, bioecological, sociocultural) prioritized by experts, and overlap these risk variables to the probability associated with changed scenarios in climate, environment, market, and demography.

**(2) Monitoring**

To identify at each scale indicators and sources of data that allow adequate monitoring in time and space and with appropriate quality (resolution, units, sensitivity, specificity, accuracy, etc.).

**(3) Early Warning**

To determine the events discriminated by scale, region, and period and for each one the field-monitoring threshold, which trigger enhanced surveillance or previously defined-validated actions of mitigation-control.

**(4) Strategy of Prevention**

To make recommendations of prevention consistent with the events already defined (risk maps, monitoring, early warning) and discriminated by scale, region and period, for example (a) microscale: peridomestic animals management, (b) mesoscale: building of dams or locality edgedeforestation for new neighborhoods, (c) macroscale, population migrations, droughts, floods.
